# Clinical Significance and Prognostic Relevance of Microsatellite Instability in Sporadic Colorectal Cancer Patients

**DOI:** 10.3390/ijms18010107

**Published:** 2017-01-06

**Authors:** Angelika Copija, Dariusz Waniczek, Andrzej Witkoś, Katarzyna Walkiewicz, Ewa Nowakowska-Zajdel

**Affiliations:** 1 Department of Nutrition Related Disease Prevention, School of Public Health in Bytom, Medical University of Silesia, 41-902 Bytom, Poland; enowakowska-zajdel@sum.edu.pl; 2 Department of Clinical Oncology, Regional Specialised Hospital No. 4 in Bytom, 41-900 Bytom, Poland; andrzejwitkos@poczta.fm; 3 Department of Propaedeutics Surgery, Chair of General, Colorectal and Polytrauma Surgery, School of Health Sciences in Katowice, Medical University of Silesia, 41-902 Bytom, Poland; dariusz_waniczek@interia.pl; 4 Department of Internal Medicine, School of Public Health in Bytom, Medical University of Silesia, 41-902 Bytom, Poland; kk.walkiewicz@gmail.com

**Keywords:** microsatellite instability, mismatch repair, colorectal cancer

## Abstract

Microsatellite instability (MSI) is a marker of the replication error phenotype. It is caused by impaired DNA mismatch repair processes (MMR), resulting in ineffectiveness of the mechanisms responsible for the DNA replication precision and postreplicative DNA repair. MSI underlies the pathogenesis of 10%–20% of colorectal cancer (CRC) cases. The data about the potential value of MMR status as a predictive factor for 5-fluorouracil (FU)-based chemotherapy remain unclear. According to National Comprehensive Cancer Network updated guidelines, MSI testing is recommended for all patients with stage II CRC because patients with MSI-H (high-frequency MSI) tumour may have a good prognosis and obtain no benefit from 5-FU-based adjuvant chemotherapy. The significance of the MSI status as a predictive factor for patients with metastatic disease was not confirmed. The association between the MSI status and the efficacy of the therapy based on anti-programmed death-1 receptor inhibitors requires further studies.

## 1. Introduction

Colorectal cancer (CRC) is a heterogeneous disease with significant differences between clinical presentation, prognosis and individual treatment response, even at the same pathological stage. CRC development is associated with various driver mutations, genetic and epigenetic tumour signatures. Epidemiological studies underline possible etiological differences according to molecular markers. All the above-mentioned factors are very important to make individually tailored treatment decisions.

Adjuvant chemotherapy based on 5-fluorouracil (5-FU) is a standard treatment for patients with node-positive CRC who underwent curative surgery. However, there is a significant group of overtreated patients who could be cured by surgery alone without adjuvant chemotherapy (about 40%) or have chemotherapy-resistant disease [1,2]. The reported five-year survival of patients with stage II CRC ranges from 75% to 87.5% [3,4,5,6] and only 3.5%–5% of this group benefits from adjuvant chemotherapy [6,7,8]. Among patients with CRC of Dukes’ stage C undergoing surgery, 5-FU-based adjuvant chemotherapy improves overall survival (OS) by 10%–12% [2].

In metastatic colorectal cancer (mCRC), 5-FU is commonly combined with oxaliplatin and/or irinotecan or trifluridine and target therapy (bevacizumab, cetuximab, panitumumab, aflibercept, ramucirumab or reforafenib). Response rates with fluorouracil-based regimens in metastatic disease reach about 25%. For 40% of mCRC patients, five-year survival could be expected due to the local and systemic therapy including tailor-made therapy based on the evaluation of the *KRAS* and *BRAF* mutation status and microsatellite instability (MSI) analysis as well [2]. Better biomarkers are needed to tailor adjuvant or palliative chemotherapy.

Microsatellite instability (MSI) is considered a promising factor that could potentially enable identification of patients that may benefit from the chemotherapy in the adjuvant schedule. The predictive value of the MSI status in palliative treatment remains controversial.

MSI is a marker of the replication error phenotype (RER) and is caused by defective DNA mismatch repair processes (MMR), resulting in loss of the mechanisms responsible for the precision of the DNA replication and postreplicative DNA repair. The frequency of mutations is increased 100- to 700-fold compared to cells with an efficient MMR system [9,10].

MSI is involved in the pathogenesis of many types of neoplasms, both hereditary and sporadic, including CRC [11,12,13,14,15,16,17,18,19], head and neck squamous cell carcinoma [20], non-small cell lung cancer [20,21], squamous and basal cell skin cancer [20], melanoma [22], gastric [23], bladder [20,24], endometrial [25,26] and ovarian cancer [25,26,27].

Approximately 85% of CRC is associated with chromosomal instability (CIN)/alterations, whereas MSI is identified in 10%–20% of all CRC cases, including sporadic CRC (12%) and hereditary non-polyposis colorectal cancer (HNPCC, Lynch syndrome; 3%) [11,12,13].

CIN in sporadic CRC in conjunction with *APC* gene mutation results in microsatellite stable (MSS), CIMP negative and *BRAF*, *KRAS* wild type tumours.

Lynch syndrome is associated with germline mutations in at least one of MMR genes, i.e., *MLH1*, *MSH2*, *MSH6* and *PMS2* and never co-exists with the BRAF mutation, while the co-existence of MMR gene defect and *KRAS* mutation is possible [28,29,30]. The presence of v-raf murine sarcoma viral oncogenes homolog B1 (*BRAF^V600E^*) excludes the diagnosis of the Lynch syndrome [29,30].

Sporadic CRCs with high-frequency MSI (MSI-H) are most often caused by epigenetic inactivation of *MLH1* gene by somatic promotor hypermethylation (about 70%–95%) while *MSH2* and *MSH6* changes are less common [11,14,15,16,17,18,19]. Unlike the Lynch syndrome, sporadic MSI CRC is associated with the *BRAF^V600E^* mutation in approximately 50% of cases [31,32]. A *BRAF* mutation is diagnosed in approximately 5%–15% of all CRC tumours and is considered a prognostic biomarker of less favourable outcome in the general population of CRC patients overall but also in dMMR subgroup—both in the early stage [30] and advanced disease [31]. The incidence of *BRAF* mutation may vary depending on tumour stage. There are reports suggesting more frequent prevalence of impaired *BRAF* mutation in deficient MMR (dMMR) mCRCs (34.6% [33]) compared to early-stage dMMR tumours (24% [31]). The increase in *BRAF* mutation in metastatic MSI cancer compared to early stage reflects a more aggressive phenotype. It seemed that the prognosis could be different in the subtypes of CRC depending on MSI/MMR, *KRAS* and *BRAF* status [29,31,34]. MSI frequency is higher among stage II CRC [33] compared to metastatic disease [35].

According to the recently described CRC classification system based on molecular characteristics of the tumour, the microsatellite instability immune type (CMS1) is one of four distinct consensus molecular subtypes [36]. CMS1 type includes MSI and/or *BRAF^V600^*^E^ tumours characterized by certain clinical and pathological features, such as more likely to be proximal, high-grade mucinous differentiation, notable lymphocyte infiltration, older age at diagnosis and female gender [32,36].

While most studies suggest higher rates of MSI in the older demographic, the results of the study conducted by Khan et al. suggested more prevalent MSI in young patients with early onset CRC (age ≤ 30 years) than in older CRC patients (age ≥ 50, *p* < 0.01) [37]. This may be explained by the higher incidence of MMR mutations and hence genetic cancers in the analysed population. According to the study, MSI presence in the young patients was not strongly associated to *MLH1*/*PMS2* loss and did not co-exist with *BRAF^V600E^* mutations (*p* < 0.01), which suggests more frequent occurrence of the Lynch syndrome in the subgroup of patients with the early onset of the disease [37].

MSI tumours are characterized by a highly upregulated expression of various immunological checkpoints that are subjects of many current clinical trials as possible therapeutic targets, i.e., PD-1, PD-L1, CTLA-4, LAG-3 or IDO [38,39,40,41,42]. The mentioned immune inhibitory signals prevent elimination of neoplastic cells by counterbalancing the active immune microenvironment of the MSI tumour [38,43]. The MSI status may be a predictive marker for immuno-modulating agents.

## 2. Microsatellite Instability (MSI) Status Assessment

According to National Comprehensive Cancer Network (NCCN) updated 2016 guidelines, screening for the Lynch syndrome is recommended for all patients with CRC diagnosed by the age of 70 years old and regardless of age if Bethesda guidelines are met [44]. Moreover, MMR or MSI testing is recommended by the NCCN for all patients with stage II CRC, based on the results obtained by Sargent et al. suggesting that patients with dMMR tumour receiving FU-based therapy may receive no benefit from adjuvant systemic therapy [7,44].

The guidelines do not refer to the assessment of MSI/MMR in patients with metastatic disease.

The updated guidelines on molecular markers for CRC are being developed jointly by the American Society for Clinical Pathology, the College of American Pathologists, American Society of Clinical Oncology and the Association for Molecular Pathology and are not yet available [45].

The MSI status can be assessed by the use of reference National Cancer Institute (NCI) panel of five microsatellite markers, including two mononucleotide repeats (BAT26 and A4725) and three dinucleotide repeats (D5S346, D2S123, and D17S250). The instability of two or more of the five markers was defined by NCI as high-frequency MSI (MSI-H), whereas instability of only one marker characterizes low-frequency MSI (MSI-L) [46].

An alternative method of MMR status evaluation is the analysis of MMR gene protein products (*MLH1*, *MSH2*, *MSH6* and *PMS2*) using the immunohistochemical (IHC) staining [32]. It is an inexpensive and increasingly available technique with high sensitivity (80%–95%) and specificity (up to 100% in most reports) [47,48,49,50]. IHC assessment of MLH1 and MSH2 protein expression has also been proven to have a high correlation degree with the MSI status estimated by polymerase chain reaction (PCR) [47].

## 3. MSI Status and Colorectal Cancer (CRC) Prognosis

Numerous studies established the value of the MSI status as a prognostic factor in all stages of CRC regardless of age [37,51,52,53]. The results of a meta-analysis including 7642 patients indicated that MSI tumours corresponded with significantly improved prognosis compared to MSS CRCs (OS associated with MSI: hazard ratio (HR) 0.65 (95% confidence interval (CI): 0.59–0.71)) [53].

There are reports suggesting a reduced rate of lymph node involvement [34,54,55,56,57,58] and distant metastases in MSI tumours compared with MSS tumours [57,58,59,60]. However, MacQuarie et al. noted no differences in the number of all lymph nodes or the count of negative lymph nodes in patients with MSI and MSS tumours in stage III [61].

Mohan et al., in a single centre study including 1250 CRC patients, noted improved disease-free survival (DFS) in patients with MSI CRCs in stage I and II disease in comparison with MSS tumours. However, in CRC patients in stage III disease, MSI tumours were characterised by more prominent lymphovascular and perineural invasion and the DFS in this subgroup was shorter than in MSS patients in stage III [57].

According to the results obtained by Kim et al. [60], patients with MSI-H CRC are more likely to have longer DFS than individuals with MSI-L or MSS tumours (HR: 0.619 (95% CI: 0.508–0.755), *p* < 0.001), more often exhibit local recurrence (30.0% vs. 12.0%, *p* = 0.032) or peritoneal metastases (40.0% vs. 12.3%, *p* = 0.003), while the extra-abdominal recurrence is less frequent compared to MSI-L/MSS CRCs (hepatic metastases: 44.7% vs. 15.0% respectively, *p* = 0.01; lung metastases 42.5% vs. 10.0%, *p* = 0.004).

On the other hand, the results obtained by Venderbosch et al. suggest an association between MSI and reduced OS in patients with advanced CRC [31]. The authors performed a pooled analysis of 4 phase III studies in the first-line treatment of metastatic CRC focusing on MMR and *BRAF* status and suggesting that the worse prognosis in dMMR mCRCs may be driven by the *BRAF* mutation. The study included 3063 patients with a low prevalence of dMMR (5%) and *BRAF* mutation (8.2%) in the whole studied population, however in patients with dMMR tumours, impaired *BRAF* was noted more often than in proficient MMR (pMMR) tumours (34.6% vs. 6.8% respectively, *p* < 0.001). Patients with MSI had significantly reduced progression-free survival (PFS; HR 1.33 (95% CI: 1.12–1.57)) and OS (HR 1.35 (95% CI: 1.13–1.61)). Interestingly, the authors observed decreased OS and PFS in mCRC patients with pMMR tumour and *BRAF* mutation while in the dMMR subgroup no association between *BRAF* status and survival was proven. On the other hand, survival effect in patients with *BRAF* mutation did not differ between pMMR and dMMR subgroup [31].

## 4. MSI Status and 5-Fluorouracil (FU) Chemotherapy Response

5-FU is an analogue of uracil and its cytotoxic activity is the cause of the misincorporation of fluoronucleotides into nucleic acids by the active metabolites of 5-FU and in the inhibition of thymidylate synthase—an enzyme taking part in the synthesis of nucleotides [62]. 5-FU cytotoxic effect seems to be determined by several enzymatic mechanisms belonging to DNA repair systems, resulting in removing of 5-FU from DNA: the base excision repair (BER; by excision of 5-FU or uracil by uracil DNA glycosylases) [63] and MMR (by removing of mismatched nucleotides) [64,65]. 5-FU and 5-fluorouracil-2′-deoxyuridine-5′-triphosphate excision from DNA may exacerbate the cytotoxicity of the drug by increasing its intracellular concentration [64].

The results of in vitro studies suggested the ineffectiveness of 5-FU in human colorectal cell lines with MSI [66,67,68]. The findings of the early clinical trials did not confirm these observations, suggesting a possible better survival in the group of patients with MSI tumours receiving 5-FU compared to the MSS CRC group [52,69,70,71]. However, it is explained by some authors as potential enhancement of better prognosis noted frequently in patients with MSI CRC [1]. Other available data suggest a potential similar benefit from 5-FU regardless of the presence of MSI or MSS [1,29,72]. On the other hand, several authors indicated a lack of benefit from 5-FU-based adjuvant therapy in patients with MSI CRC [7,73,74,75,76,77,78,79], including the suggestion that the presence of MSI may be associated with shorter survival [7,75]. The data about the potential value of the MMR status as a predictive factor for 5-FU-based adjuvant chemotherapy are contradictory.

Guastadisegni et al. [64] conducted a meta-analysis in order to assess the clinical significance of the MSI status for CRC patients receiving 5-FU-based chemotherapy. The study included 12,782 patients with various tumour stages examined in 31 studies reporting survival data. Authors confirmed the association between MSI and improved outcome in terms of DFS (summary OR of 0.58 (95% CI: 0.47–0.72), *p* < 0.0001) and OS (summary OR of 0.6 (95% CI: 0.53–0.69), *p* < 0.0001) irrespective of the tumour stage. The analysis of the potential survival effect of 5-FU adjuvant chemotherapy depending on the MSI status was based on the evidence from seven studies in which survival data were stratified separately for MSI-H and MSS patients. MSS patients have been shown to obtain benefit from 5-FU-based chemotherapy, whereas the survival effect in the MSI group did not reach statistical significance. The statistical analysis showed the high inter-study heterogeneity in the MSI subgroup. The possible explanation may lie in a low number of sporadic MSI-H tumours (396 MSI vs. 2467 MSS patients in the analysed population) [64].

Webber et al. [80] performed another meta-analysis on this topic including 9312 patients in all CRC stages reported in 16 studies, of which 10 were previously analysed by Guastadisegni et al. Not surprisingly, the authors found a beneficial effect of 5-FU-based chemotherapy among patients with MSS tumours based on DFS (HR of 0.62 (95% CI: 0.54, 0.71)) and OS (HR of 0.65 (95% CI: 0.54, 0.79)). Although the study included a larger group of MSI-H patients compared to Guastadisegni’s paper (*n* = 1293 vs. 396 MSI), still due to statistically significant heterogeneity in the MSI-H group no clear survival effects could be observed (DFS in MSI-H group: HR of 0.84 (95% CI: 0.53, 1.32); OS: HR 0.66 (95% CI: 0.43, 1.03)).

## 5. Predictive Value of MSI Status in II and III CRC

According to the NCCN guidelines, MSI testing is recommended for all patients with stage II CRC, which is based on the assumption that these individuals obtain no benefit from 5-FU-based adjuvant therapy [44] based on the results obtained by Sargent et al. [7]. Unlike individuals with MSS tumours, patients with stage II and III MSI CRC did not benefit from 5-FU chemotherapy compared to patients undergoing surgical treatment alone (DFS: HR 1.10 (95% CI: 0.42–2.91)). The data were pooled with further 570 cases from a previous study [75]. In the combined dataset, in patients with MSI CRC in stage II, adjuvant chemotherapy was associated with decreased OS (HR 2.95 (95% CI: 1.02–8.54)).

Taking into account that the NCCN recommendations are based on one study and considering the finding of the two large meta-analyses by Guastadisegni et al. and Webber et al., the substantiation of MSI status determination in stage II CRC patients may be controversial. However, Des Guetz et al. [81] undertook a meta-analysis focusing on patients with stage II and III CRC. Seven studies and 3690 patients were taken into account, including 810 patients with tumour stage II and 2444 with stage III. Six of seven included studies were analysed by Guastadisegni et al. [64]. Des Guetz et al. observed no statistically significant difference in patient survival in terms of Regression-Free Survival (RFS; HR 0.96 (95% CI: 0.62–1.49)) or OS (HR 0.70 (95% CI: 0.44–1.09)) irrespective of adjuvant chemotherapy implementation for the MSI-H patients. Moreover, MSI-H patients in the study obtained the minor benefit from treatment compared to the MSS group (RFS: HR 0.77 (95% CI: 0.67–0.87)). Thus, the authors indicated the MSI status as an encouraging predictive factor of non-response to 5-FU-based adjuvant chemotherapy in stage II or III CRC.

## 6. Predictive Value of MSI Status in Metastatic CRC

There are insufficient data about the significance of MSI in the mCRC patients. Most of the available studies were conducted without randomization, based on small groups and without clear discrimination of sporadic and hereditary CRCs [35]. It may be an effect of a decreased metastatic potential of MSI tumours [57,58,59] resulting in a reduced incidence of MSI CRC among patients with advanced disease [58].

A meta-analysis undertaken by Des Guetz et al. [82] focused on potential predictive significance of MSI assessment for patients with metastatic disease. The analysis included 6 studies representing 964 patients receiving 5-FU-based chemotherapy (30%) or combinations of 5-FU or capecitabine with oxaliplatin and/or irinotecan (70%). While OS and RFS data were unavailable, the parameter used to assess benefit of chemotherapy was response rate (RR) ratio according to the RECIST criteria. No significant difference in effect of treatment in terms of RR ratio was observed for the MSI-H compared with the MSS patients (RR ratio 0.82 (95% CI: 0.65–1.03; *p* = 0.09)). The possible cause may be a considerable statistically significant heterogeneity probably arising from different chemotherapy regiments and an unequal distribution of patients in the studies.

A recent phase II study by Le et al. [43] evaluated the efficacy of anti-programmed death 1 (PD-1) receptor inhibitor pembrolizumab depending on the MSI status. The analysed population consisted of 41 patients with progressive metastatic disease, including 13 dMMR CRC, 25 pMMR CRC and 10 MMR-deficient non-colon cancer. The immune-related objective RR and 20-week-immune-related PFS rate were 40% (95% CI: 12%–74%) and 78% (95% CI: 40%–97%) respectively, for dMMR CRC and 0% (95% CI: 0%–20%) and 11% (95% CI: 1%–35%) for pMMR CRCs. The median PFS and OS in the pMMR CRC cohort were 2.2 and 5.0 months, respectively, while in the dMMR CRC group PFS and OS have not been reached yet. The results suggest that patients with dMMR tumours are more responsive to pembrolizumab than pMMR mCRC patients. However, the results need confirmation in further well-designed studies in a larger population.

On the other hand, Kim et al. [60] analysed the outcomes in patients with colorectal cancer recurrence depending on the MSI status. The study group included 2940 patients with stage I–II CRC after curative resection. MSI-H was observed in 8.9%. Although MSI-H was associated with significantly longer OS in patients without recurrence compared to MSI-L or MSS, among individuals with recurrence MSI-H CRCs was associated with worse OS from diagnosis to death (HR: 1.363 (95% CI: 1.022–1.819), *p* = 0.035) and OS from recurrence to death (HR: 2.667 (95% CI: 1.541–4.616), *p* < 0.001). The most frequent treatment strategy after recurrence in patients with MSI-H tumours was palliative chemotherapy without resection (74.0% compared to 44.1% in MSI-L/MSS patients, *p* = 0.01). Patients in this group underwent reoperation less often compared to MSI-L/MSS patients with recurrence (20.0% vs. 51.6%, *p* = 0.01). However, there was a major disproportion between the number of MSI-H and MSI-L/MSS patients with CRC recurrence (20 vs. 374 patients, respectively).

The main characteristic of available meta-analysis on prognostic value of microsatellite instability (MSI) are summarised in Table 1.

## 7. Conclusions

Colorectal cancer is a heterogeneous disease with varied biological and genetic features. The heterogeneity of CRC leads to different prognosis and clinical management. Treatment decisions should be based in each case individually on various clinical factors, including driver mutations, and genetic and epigenetic signatures identified in the tumour.

MSI is a promising factor that could potentially enable predicting patient response to the treatment based on the reports postulating no benefit from adjuvant 5-FU chemotherapy in patients with stage II and III CRC if MSI was detected. MSI may also be associated with favourable prognosis and a better overall survival in patients with advanced disease. However, the role of the MSI status as a predictive marker remains controversial.

In our opinion, the individually tailored treatment decision and the consideration of more aggressive approach may be needed in the *BRAF* mutated tumours and alternative chemotherapy in the MSI tumours.

The preliminary studies on the potential role of MSI in targeted immunotherapy have been promising and further investigation on this topic is warranted.

## Figures and Tables

**Table 1:** Characteristic of available meta-analysis on prognostic and predictive value of microsatellite instability (MSI).

Study, Year	Number of Included Trials	Number of Patients	CRC Stage	Received Treatment	MSI Status	Main Findings	Predictive Value of MSI Status
Popat et al., 2005 [53]	32	7642	I–IV	5-FU-based adjuvant chemotherapy vs. control group	1277 MSI	MSI-H status established as a prognostic factor; no benefit from adjuvant FU for MSI patients	Not assessed
Guastadisegni et al., 2010 [64]	31	12,782	I–IV	5-FU-based adjuvant chemotherapy in combination with levamisole or leucovorin (in 6 studies) or mitomycin (in 1 study)	14% MSI (396 MSI, 2467 MSS)	MSI-H status established as a prognostic factor (association between MSI and favourable prognosis in term of OS and DFS; inconclusive results about predictive value of MSI status due to the high inter-study heterogeneity	Inconclusive results
Webber et al., 2015 [80]	16	9312	I–IV	5-FU-based chemotherapy vs. control group	15% MSI	No difference in the effect of treatment based on MSI status	Not proven
Des Guetz et al., 2009 [81]	7	3690	II–III	5-FU-based adjuvant chemotherapy vs. control group	14% MSI (454 MSI-H; 3690 MSS)	MSI-H status established as a predictive factor of non response to 5-FU-based chemotherapy in CRC patients stage II or III	Proven for patients stage II/III
Des Guetz et al., 2009 [82]	6	964	IV	5-FU-based chemotherapy or combinations of 5-FU or capecitabine with oxaliplatin and/or irinotecan	9% MSI (91 MSI-H, 873 MSS)	No difference in the effect of treatment of patients with metastatic CRC in terms of RR based on MSI status	Not proven for mCRC patients

CRC, colorectal cancer; FU, fluorouracil; MSS, microsatellite stable; MSI-H, high-frequency MSI; OS, overall survival; DFS, disease free survival; RR, response rate; mCRC, metastatic colorectal cancer.
